# Social and behavioural determinants of syphilis: Modelling based on repeated cross-sectional surveys from 2010 and 2017 among 278,256 men who have sex with men in 31 European countries

**DOI:** 10.1016/j.lanepe.2022.100483

**Published:** 2022-08-09

**Authors:** Ana Mendez-Lopez, David Stuckler, Ulrich Marcus, Ford Hickson, Teymur Noori, Robert N. Whittaker, Klaus Jansen, Asuncion Diaz, Lukasz Henszel, Annie Velter, Jan C. Semenza, Axel J. Schmidt

**Affiliations:** a Department of Preventive Medicine, Public Health, and Microbiology, School of Medicine, Autonomous University of Madrid, Spain; b Dondena Research Centre, Bocconi University, Italy; c Department for Infectious Disease Epidemiology, Robert Koch Institute, Berlin, Germany; d Sigma Research, Department of Public Health, Environments & Society, London School of Hygiene & Tropical Medicine, London, United Kingdom; e European Centre for Disease Prevention and Control, Stockholm, Sweden; f Section for respiratory, blood-borne and sexually transmitted infections, Department of Infection Control and Vaccines, Norwegian Institute of Public Health, Norway; g National Centre of Epidemiology, Instituto de Salud Carlos III. CIBER de Enfermedades Infecciosas (CIBERINFEC), Spain; h Department of Infectious Disease Epidemiology and Surveillance, National Institute of Public Health - National Institute of Hygiene, Warsaw, Poland; i Public Health Protection Unit, NHS Greater Glasgow and Clyde, Glasgow, United Kingdom; j Health Promotion and Prevention Division, Santé Publique France, Saint Maurice, France; k Heidelberg Institute of Global Health, University of Heidelberg, Germany

**Keywords:** Men who have sex with men, MSM, Homosexual, Syphilis, Europe, STI-screening, Pre-exposure prophylaxis, PrEP, Condomless anal intercourse, Survey

## Abstract

**Background:**

Syphilis case notifications among men-who-have-sex-with-men (MSM) have increased markedly over the past two decades in Europe. We tested several potential factors for this resurgence.

**Methods:**

Self-reported data from two cross-sectional waves of the European MSM Internet Survey (EMIS-2010 and EMIS-2017, N = 278,256 participants living in 31 European countries) were used to fit multivariable hierarchical logistic regression models designed to evaluate potential social, behavioural, and interventional determinants of syphilis diagnosis. Additional multivariable hierarchical negative binomial models investigated determinants of the number of non-steady male condomless anal intercourse (CAI) partners. We tested the hypothesis that more CAI and syphilis-screening are associated with syphilis resurgence, both linked to use of pre-exposure prophylaxis (PrEP).

**Findings:**

Between 2010 and 2017, incidence of syphilis diagnosis in the previous 12 months rose from 2.33% (95%CI: 2.26–2.40) of respondents reporting a syphilis diagnosis in 2010 compared with 4.54% (95%CI: 4.42–4.66) in 2017. Major factors contributing to syphilis diagnosis were living with diagnosed HIV (adjusted odds ratio (aOR) 2.67, 95%CI: 2.32–3.07), each additional non-steady male CAI partner (aOR 1.01, 95%CI: 1.01–1.01), recency of STI-screening (previous month vs no screening, aOR 25.76, 95%CI: 18.23–36.41), selling sex (aOR 1.45, 95%CI: 1.27–1.65), and PrEP use (aOR 3.02, 95%CI: 2.30–3.96). Living with diagnosed HIV (adjusted incidence rate ratio (aIRR) 3.91, 95%CI: 3.77–4.05), selling sex (aIRR 4.39, 95%CI: 4.19–4.59), and PrEP use (aIRR 5.82, 95%CI: 5.29–6.41) were associated with a higher number of non-steady male CAI partners. The association between PrEP use and increased chance of syphilis diagnosis was mediated by STI-screening recency and number of non-steady male CAI partners, both substantially higher in 2017 compared to 2010.

**Interpretation:**

Syphilis cases are concentrated in three MSM population groups: HIV-diagnosed, PrEP users, and sex workers. Behavioural and interventional changes, particularly more non-steady male CAI partners and recency of STI-screening, are major contributing factors for increasing syphilis diagnoses among MSM in Europe.

**Funding:**

European Centre for Disease Prevention and Control.

**Research in context**

**Evidence before this study**

We searched PubMed for articles in any language between 1 January 2000 and 31 January 2022 using the following keywords and related terms in the title or abstract, or as MeSH terms, if existing: ‘syphilis’, ‘men who have sex with men’, and ‘Europe’, to identify individual studies and reviews. We additionally assessed surveillance data reports and found reports from the European Centre for Disease Prevention and Control showing increasing trends of syphilis case notifications to be concentrated in MSM. We found a recent systematic review and meta-analysis reporting pooled syphilis prevalence among MSM in Europe and North America in 2010-2020 was twice as high as in the previous decade 2000-2009, whereby the pooled prevalence has risen from 2.1% (95% CI: 0.8 to 3.9) in the period 2000-2009 to 4.2% (95% CI: 1.7 to 7.6) in 2010-2020. Another systematic review of syphilis trend studies in Western Europe and North America also reports increases in diagnoses since the year 2004. Several country-level studies show increases in syphilis case notifications, incidence or prevalence among MSM in different European countries. Some of these country studies assess determinants of syphilis diagnosis, including changes in sexual practices, sexual networks, and sexual healthcare contexts. Although there is evidence on the increasing trends of syphilis among MSM in Europe, there was limited direct evidence assessing simultaneously social, behavioural, and interventional determinants cross-nationally.

**Added value of this study**

This is the largest multi-country study documenting a higher rate of syphilis diagnoses between two time points (2010 and 2017) using data from repeated cross-sectional surveys among MSM in Europe along with the assessment of social, behavioural, and interventional determinants using harmonised survey data across countries. Incidence of syphilis in the MSM population has risen across Europe. Syphilis cases are concentrated in three MSM population groups: MSM diagnosed with HIV, MSM using PrEP, and MSM selling sex. The rise of syphilis diagnoses has disproportionately impacted HIV-diagnosed MSM and MSM sex workers. Major determinants are recency of last asymptomatic screening and number of non-steady condomless anal intercourse (CAI) male partners, both higher in 2017 compared to 2010, and both factors mediating the association between PrEP use and higher chance of syphilis diagnosis.

**Implications of all the available evidence**

MSM disclosing multiple CAI partners should be offered regular syphilis-screening due to their clearly increased risk and the serious possible sequelae of syphilis. Guidelines for the management of patients using PrEP include frequent syphilis-screening. Our finding that over a third of PrEP users were screened for STIs within the previous month provides support for the feasibility of implementing a regular HIV/STI-screening approach, such as the one in European guidelines for the management of PrEP users. Further, community-based education in MSM communities is needed to increase knowledge of and social norms for syphilis-screening. Efforts should be made to increase STI-screening in the MSM population, particularly among MSM sub-populations (HIV-diagnosed and sex workers) at high risk for syphilis, at least to match the sub-population with the highest screening rates (PrEP users).

Alt-text: Unlabelled box

## Introduction

Syphilis is a curable sexually transmitted infection caused by *Treponema pallidum*, a motile Gram-negative spirochaete. Syphilis case notifications have risen markedly in many western European countries since record lows in 1998.^1^, ^2^, ^3^ German surveillance data reveal a consistent increase in cases from 1955 in 2001 to 4077 in 2010, then to 7396 in 2020.^4^ Similar patterns have been observed, among others, in England and Wales,^5^ Scotland,^6^ France,^7^ and Norway.^8^ A disproportionate burden of increasing syphilis diagnoses is occurring among MSM, of whom many have HIV co-infection.^1^, ^2^, ^3^^,^^9^, ^10^, ^11^, ^12^ This rise in syphilis is a sharp reversal of trends in the 1990s, when rates stabilized or declined in most European countries and the United States.^12^

The reasons for the resurgence of syphilis among MSM in Europe are not well understood. Increases in syphilis diagnoses in the United States and western Europe have been attributed to multiple behavioural factors, including changes in sexual practices (*e.g*., increase in condomless anal intercourse (CAI), serosorting, the use of stimulant drugs before or during sex (chemsex), transactional sex, multiple (often anonymous) sexual partners), expansion of sexual networks (facilitated by technological developments such as the internet and geospatial apps for finding partners), and changes in sexual healthcare contexts (*e.g.,* increased care-seeking behaviour through uptake of STI-screening and use of chemotherapies).^3^^,^^11^, ^12^, ^13^, ^14^, ^15^, ^16^, ^17^, ^18^ Hypothesised drivers for these changes include elimination of HIV infectivity in HIV positive men on treatment and elimination of HIV susceptibility through chemoprophylaxis (in particular PrEP) in HIV-negative men.^12^^,^^19^^,^^20^

Further, rising syphilis rates have not been curbed, in part, due to the insufficient scale of prevention services as part of syphilis programmes, including better performance in active case-finding and curing cases, healthcare workers awareness, laboratory capacity and healthcare infrastructure, and funding.^12^^,^^19^ Coverage of preventive healthcare services has been particularly problematic in populations with high incidence of STI such as MSM, for whom coverage of services such as case finding may be particularly problematic as stigmatised sexual minority and sexual settings with a high degree of anonymity.

Syphilis is a notifiable disease in all EU/EFTA countries, with reliable surveillance data based on laboratory-confirmed diagnosis. However, gaps exist in the surveillance of syphilis in the MSM population. For example, syphilis surveillance data in some EU countries are not disaggregated by gender of sexual partners,^21^ making it difficult to compare trends across countries and over time. The European MSM Internet Survey (EMIS) is a community-recruited, self-selecting, self-reporting online cross-sectional survey for MSM. It combines epidemiological, psychosocial, behavioural, and interventional data, and is the largest dataset of this type across European countries. Surveys were conducted in 2010 and 2017.^22^, ^23^, ^24^, ^25^ Using EMIS data, we examined some potential determinants of rising syphilis rates among MSM in this study period. We investigated social, behavioural, and interventional factors linked to the MSM syphilis epidemic, testing the hypothesis that an increase in CAI with non-steady partners is associated with syphilis resurgence, and examining factors that may be associated with the number of CAI partners, including HIV PrEP use and associated more frequent screening detecting more syphilis cases.^26^

## Methods

### Source of data

Details of the EMIS survey have been described elsewhere.^24^^,^^25^ Briefly, a non-probability sample of participants was recruited through direct-to-user invitations in online-dating platforms, geo-spatial dating apps (2017 only), and social media channels, and ads on websites of EMIS civil society partners (more details in Appendix 1 and on the project's website: www.emis2017.eu). EMIS-2010 was available online for completion for 12 weeks, between 4 June and 31 August 2010.^22^ Online promotion of EMIS-2017 began on 18 October 2017 and ran until 31 January 2018.^23^ For these analyses we included respondents from 30 European countries that as of 2017 were part of the European Centre for Disease Prevention and Control mandate on disease surveillance. We also included respondents from Switzerland and four European microstates (Andorra, Monaco, San Marino, and Liechtenstein, all of which are included in the samples of neighbouring countries). Hence in this publication we refer to 31 European countries, while technically respondents from 35 countries are included in the study sample.

### Main outcome measure

Syphilis diagnosis was self-reported. Participants were asked if they had ever been diagnosed with syphilis and, if so, when they had last been diagnosed (within 24 hours, last 7 days, 4 weeks, 6 months, 12 months, 5 years, or longer ago). We constructed the incidence of syphilis diagnosis based on self-reported diagnosis within the previous 12 months.

Respondents using the French version of the 2017 questionnaire were likely to over-report syphilis diagnoses because of sub-optimal translation of questions on STI diagnoses (details in Appendix 1).^23^ All statistical models are adjusted for potential bias arising from this issue.

### Exposure variables

We assessed five sociodemographic variables as determinants of syphilis diagnosis and number of non-steady male CAI partners, including age (and age squared to account for potential non-linear effects of age during the life course), educational level, occupational status, settlement size, and whether the respondent was born in the country of residence.

To capture risky sexual behaviour with respect to syphilis transmission, we assessed the number of male sexual partners by type and sexual act within the previous 12 months, differentiating whether partners were steady or non-steady, and whether the sexual act included condomless anal intercourse (CAI).^27^ Additional behavioural risk factors are whether the respondent engaged in transactional sex, including paying for sex and selling sex during the previous 12 months.

We assessed care-seeking behaviour for STI-screening (other than HIV) as the recency of last screening. Thus, between survey waves, recency of the last STI-screening is a marker for behavioural/interventional change. Additionally, recency of screening is a marker of uptake of and adherence to PrEP guidelines, thus, being a factor affecting the likelihood of diagnosing syphilis, particularly recent and asymptomatic infections. STI-screening almost universally featured a blood test in both waves.^26^

We also assessed HIV-serosorting (whether the respondent had CAI only with males with the same HIV diagnosis as himself), knowledge that an undetectable HIV viral load equals untransmissibility (U=U), HIV diagnosis, and whether the respondent used PrEP daily or on demand or not. PrEP was not established/available in 2010 and thus captured only in the 2017 wave. Analyses involving this variable are restricted to data for only the second wave.

Finally, we accounted for the potential role of respondents’ survey recruitment source, differentiating between recruitment via dating apps, social media, or unknown (Supplemental data: Appendix 1).

### Statistical analyses

First, we report estimates of incidence of self-reported syphilis diagnosis within the previous 12 months in 31 European countries for the years 2010 and 2017, estimating the overall change between survey waves adjusted for whether the language of the questionnaire was French.

Second, individual-level multivariable hierarchical logistic regression models (generalized linear models with logit-link function and binomial distribution) with country random intercepts were used to examine associations with the odds of syphilis diagnosis. Next, we ran additional individual-level multivariable hierarchical negative binomial models (generalized linear model with log-link and negative binomial distribution) with country random intercepts to estimate determinants of the incidence rate for the number non-steady male CAI partners. Finally, subsequent hierarchical negative binomial and logistic regression models with country random intercepts examine, respectively, the association of PrEP use with the incidence rate of the number of non-steady male CAI partners and odds of syphilis diagnosis, testing for mediation of number of non-steady male CAI partners and recency of last STI-screening, as proxy of disease detection through testing frequency, in the association between PrEP use and the outcome measure in 2017. Estimates from regression models were used to compute marginal mean probabilities of syphilis diagnosis and mean number of non-steady male CAI partners.

In further models we assessed robustness to model specification. We report robust standard errors clustered by country. Missing data were handled with pairwise deletion. Analyses were performed using Stata 16.0.^28^

### Role of the funding source

The funder of the study had no role in study design, data collection, data analysis, data interpretation, or writing of the report. The lead author had full access to all the data in the study and had final responsibility for the decision to submit for publication.

## Results

The final analytic sample, after excluding cases with missing answers to the main outcome measure, included 166,426 (2010 wave) and 111,830 (2017 wave) people identifying as men who have sex with men and/or being sexually attracted to men (N = 278,256).

### Change in the incidence of syphilis diagnosis

**Figure 1:**
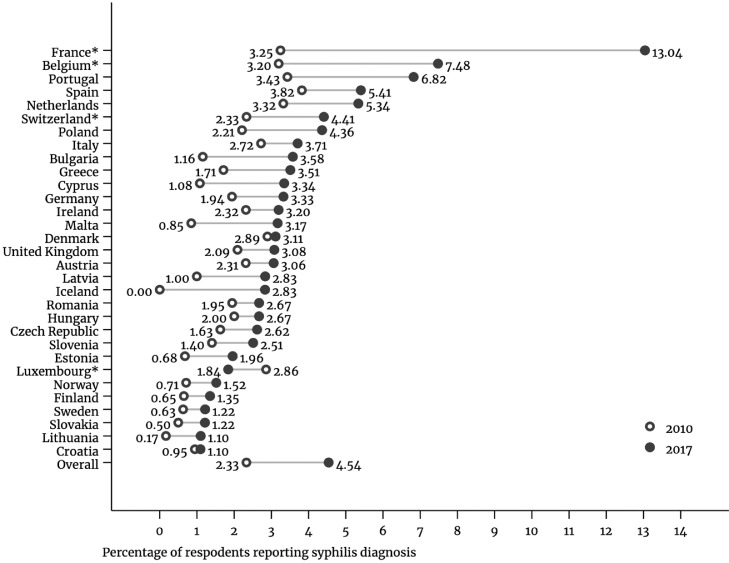
Incidence of self-reported syphilis diagnosis within the previous 12 months in 31 European countries, European Men-who-have-sex-with-men Internet Survey: EMIS-2010 and EMIS-2017. * Country with a high proportion of respondents using the French version of the questionnaire likely to have inflated the total number of affirmative responses to syphilis diagnoses in 2017 because of how the question was asked (sub-optimal translation); percentage does not exclude responses from those who used the French questionnaire.

In 2010, 3875 (2.33%, 95%CI: 2.26–2.40) respondents reported a syphilis diagnosis in the previous 12 months compared with 5074 (4.54%, 95%CI: 4.42–4.66) in 2017 (Figure 1 and Table 1), a difference of 2.21 percentage points (ppt) (95% confidence interval (CI): 2.08–2.34), which dropped to 1.37ppt (95%CI: 1.12–1.62) after adjusting for French language questionnaire and country fixed effects (Supplemental data: Appendix 2).

**Table 1:** Summary statistics in 31 European countries^a^, European Men-who-have-sex-with-men Internet Survey: EMIS-2010 and EMIS-2017.

	N	Mean (SD) or proportion	N	Mean (SD) or Proportion
	2010		2017	
**Sample size**^a^	166,426	100%	111,830	100%
**Syphilis diagnosis previous 12 months**				
No	162,551	97.67%	106,756	95.46%
Yes	3875	2.33%	5074	4.54%
**Age**	166,426	34.34 (11.37)	111,830	37.99 (12.95)
**Educational level**				
Low	13,987	8.45%	5220	5.02%
Mid at least upper secondary; 2–5 years post 16	71,197	43.02%	37,725	36.31%
High first stage of tertiary or more; 6+ years post 16	80,331	48.53%	60,955	58.67%
**Occupational status**				
Employed (full/part/self)	117,903	71.14%	80,734	72.39%
Unemployed	10,062	6.07%	5977	5.36%
Student	25,752	15.54%	15,648	14.03%
Retired, long-term sick leave, other	12,018	7.25%	9163	8.22%
**Settlement size**				
Village/small town <100,000 inhabitants	55,529	34.19%	38,785	35.07%
Big/medium town ≥100,000 inhabitants	106,884	65.81%	71,807	64.93%
**Country of birth**				
Born in country of residence	140,666	86.66%	96,320	86.24%
Born abroad	21,662	13.34%	15,366	13.76%
**HIV diagnosis**				
No	152,364	92.05%	99,605	89.64%
Yes	13,158	7.95%	11,511	10.36%
**STI-screening**				
No STI-screening previous 12 months	106,020	67.80%	58,743	54.59%
Last STI-screening during previous month	7032	4.50%	9194	8.54%
Last STI-screening 2–6 months ago	18,242	11.67%	19,696	18.30%
Last STI-screening 7–12 months ago	13,658	8.73%	10,731	9.97%
Symptomatic STI test during previous 12 months	11,415	7.30%	9244	8.59%
**Number of steady male sexual partners in the previous 12 months**	165,287	1.82 (5.95)	110,868	1.59 (5.53)
**Number of non-steady male sexual partners in the previous 12 months**	164,538	11.25 (25.04)	110,136	12.69 (26.56)
**Number of condomless intercourse steady male partners in the previous 12 months**	164,183	0.68 (2.95)	108,596	0.83 (3.73)
**Number of condomless non-steady male partners in the previous 12 months**	162,562	1.75 (9.86)	107,231	3.73 (14.93)
**Paid for sex in the previous 12 months**				
No	153,249	92.81%	101,590	90.88%
Yes	11,873	7.19%	10,200	9.12%
**Sold sex in the previous 12 months**				
No	157,356	95.24%	107,000	95.75%
Yes	7867	4.76%	4752	4.25%
**Serosorting in the previous 12 months**^c^				
No	126,104	92.04%	88,798	90.45%
Yes	10,902	7.96%	9379	9.55%
**Knowledge of U=U**				
I didn't know/understand/believe/wasn't sure	82,806	49.90%	45,762	41.02%
I knew this already	83,151	50.10%	65,809	58.98%
**PrEP use**^b^				
Not currently taking PrEP	-	-	96,305	96.71%
Currently taking PrEP daily or on demand	-	-	3281	3.29%

a Some countries include the responses of neighbouring smaller states with low number of respondents. List of microstates (with name of larger state with which their data is merged in brackets): Monaco (France), San Marino (Italy), Andorra (Spain), and Liechtenstein (Switzerland).

b Remaining number of participants are HIV-diagnosed individuals not eligible for PrEP use.

c Non-steady male CAI partners based on HIV-serosorting in the previous 12 months.

Figure 1 plots the estimated incidence for the previous 12 months by country. Portugal (3.39%, 95%CI: 2.41–4.38), Bulgaria (2.42%, 95%CI: 1.13–3.71), and Malta (2.32%, 95%CI: 1.03–5.68) had the largest estimated difference in reported incidence between 2010 and 2017 (excluding the countries with high proportions of respondents using the French questionnaire), whereas Sweden (0.59%, 95%CI: 0.14–1.04), Denmark (0.22%, 95%CI: 0.92–1.36) and Croatia (0.15%, 95%CI: 0.92–1.22) had the smallest. Only Luxembourg had a lower proportion of respondents reporting a syphilis diagnosis in 2017 compared with 2010, although not statistically significant (-1.02%, 95%CI: -4.04–2.00).

### Determinants of syphilis diagnosis

The odds of syphilis diagnosis in the previous 12 months increased with age (aOR 1.05 per year of age, 95%CI: 1.03–1.07) (Table 2). We found a dose-response relationship in the association between the odds of syphilis diagnosis and educational level, whereby a higher educational level was associated with lower odds (aOR high vs low level: 0.64, 95%CI: 0.56–0.72; aOR medium vs low level: 0.79, 95%CI: 0.70–0.91). Compared to employed individuals, unemployed individuals had higher odds of diagnosis (aOR 1.12, 95%CI: 1.04–1.20), while students (aOR 0.75, 95%CI: 0.70–0.81) and individuals retired, on long-term sick leave or other (aOR 0.86, 95%CI: 0.77–0.97) had lower odds of syphilis diagnosis. No difference in odds was found between individuals living in different size settlements (aOR 0.94, 95%CI: 0.85–1.04). Individuals born abroad their country of residence were 1.22 times more likely of being diagnosed with syphilis (95%CI: 1.10–1.36).

**Table 2:** Determinants of change in the odds of reporting a syphilis diagnosis within the previous 12 months in 31 European countries, European Men-who-have-sex-with-men Internet Survey: EMIS-2010 and EMIS-2017.

	Syphilis diagnosis	
	Adjusted odds ratio	95% confidence interval
**Year**		
2010	reference	
2017	1.181^⁎⁎^	1.062 to 1.313
**Questionnaire language**		
Other than French	reference	
French	2.837^⁎⁎⁎^	2.374 to 3.390
**Age**	1.051^⁎⁎⁎^	1.033 to 1.070
**Age squared**	0.999^⁎⁎⁎^	0.999 to 0.999
**Educational level**		
Low	reference	
Mid at least upper secondary; 2–5 years post 16	0.794^⁎⁎⁎^	0.695 to 0.907
High first stage of tertiary or more; 6+ years post 16	0.635^⁎⁎⁎^	0.561 to 0.719
**Occupational status**		
Employed full/part/self	reference	
Unemployed	1.117^⁎⁎^	1.039 to 1.201
Student	0.751^⁎⁎⁎^	0.695 to 0.812
Retired/Long-term sick leave/Other	0.863*	0.772 to 0.966
**Settlement size**		
Small town/village <100,000 inhabitants.	reference	
Medium/big town ≥100,000 inhabitants	0.940	0.848 to 1.043
**Country of birth**		
Born in country of residence	reference	
Born abroad	1.221^⁎⁎⁎^	1.099 to 1.357
**Diagnosed with HIV**		
No	reference	
Yes	2.669^⁎⁎⁎^	2.321 to 3.068
**Recency of last screening or testing**		
No STI-screening previous 12 months	reference	
Last STI-screening during previous month	25.767^⁎⁎⁎^	18.233 to 36.414
Last STI-screening 2–6 months ago	16.061^⁎⁎⁎^	11.630 to 22.180
Last STI-screening 7–12 months ago	7.250^⁎⁎⁎^	5.372 to 9.784
Symptomatic STI test during previous 12 months	74.815^⁎⁎⁎^	51.443 to 108.807
**Number of steady male sexual partners in the previous 12 months**	1.006*	1.001 to 1.011
**Number of non-steady male sexual partners in the previous 12 months**	1.004^⁎⁎⁎^	1.002 to 1.005
**Number of steady male CAI partners in the previous 12 months**	1.006	0.999 to 1.013
**Number of non-steady male CAI partners in the previous 12 months**	1.008^⁎⁎⁎^	1.007 to 1.009
**Paid for sex in the previous 12 months**		
No	reference	
Yes	1.223^⁎⁎⁎^	1.121 to 1.335
**Sold sex in the previous 12 months**		
No	reference	
Yes	1.446^⁎⁎⁎^	1.267 to 1.650
**Survey recruitment source**		
Dating apps (Romeo, Grindr, Hornet, other dating apps/websites)	reference	
Social media (Facebook, Twitter, Instagram, other)	0.814^⁎⁎⁎^	0.740 to 0.895
Unknown tracking code	0.909	0.770 to 1.073
Country random intercepts	1.141^⁎⁎^	1.053 to 1.236
Number of individuals	234719	

* *p* < 0.05, ^⁎⁎^*p* < 0.01, ^⁎⁎⁎^*p* < 0.001; robust standard errors adjusted by country; CAI, condomless anal intercourse.

**Figure 2:**
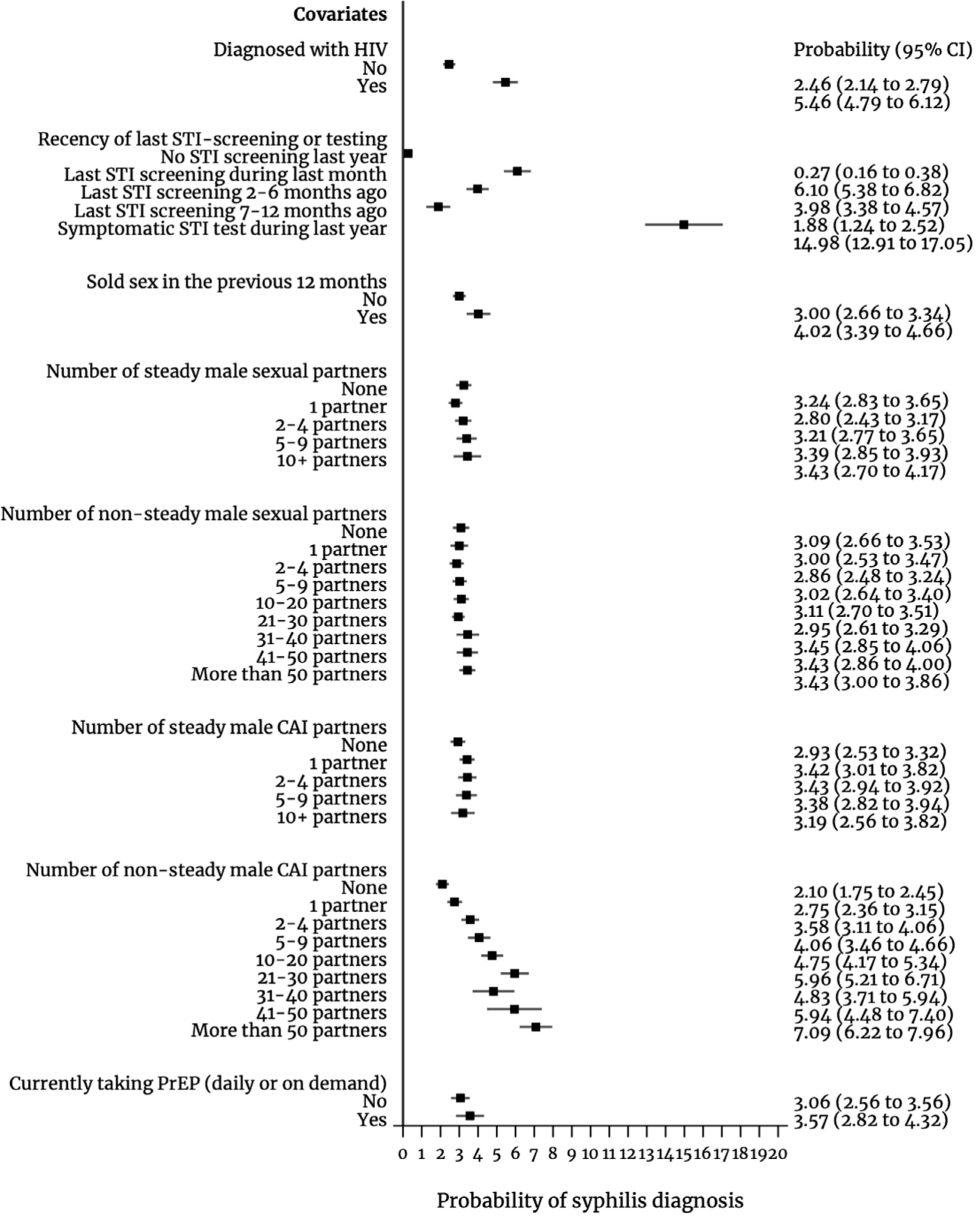
Predicted probability of syphilis diagnosis for selected covariates (marginal predicted mean probability) in 31 countries, European Men-who-have-sex-with-men Internet Survey: EMIS-2010 and EMIS-2017. CAI: condomless anal intercourse. Results for covariate measuring PrEP use are only for the year 2017 and the estimates for this covariate are based on a sample size of only 30 countries as in one country (Latvia) there were no PrEP users.

**Figure 3:**
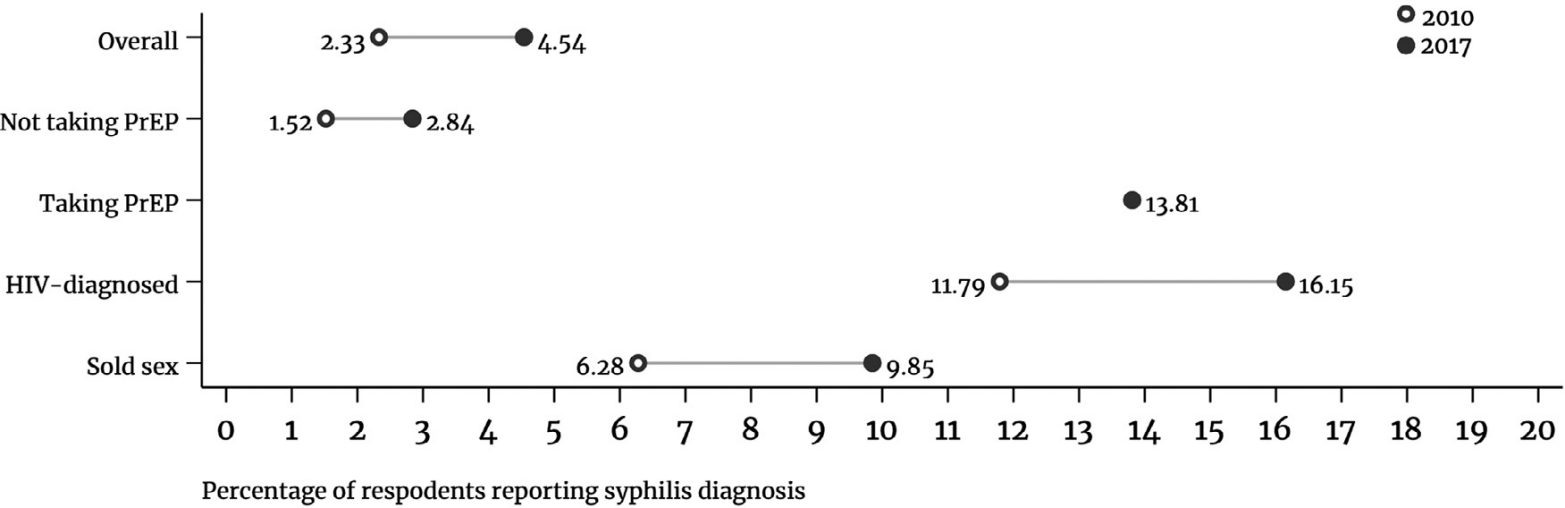
Incidence of self-reported syphilis diagnosis within the previous 12 months overall and in key population groups across 31 European countries, European Men-who-have-sex-with-men Internet Survey: EMIS-2010 and EMIS-2017. *Note*: ‘Not taking PrEP’ sample in 2010 includes the overall sample except for individuals diagnosed with HIV who would have not been eligible for PrEP. In 2017, ‘not taking PrEP’ sample includes the overall sample except for individuals diagnosed with HIV who are not eligible for PrEP and individuals taking PrEP.

Living with diagnosed HIV was associated with higher odds of syphilis diagnosis (aOR 2.67, 95%CI: 2.32–3.07) (Table 2), which accounted for a 5.46% marginal mean probability of syphilis diagnosis among individuals living with diagnosed HIV and 2.46% probability among individuals not living with diagnosed with HIV (Figure 2). Across survey waves, the proportion of respondents living with HIV reporting a syphilis diagnosis (13.82%, 95%CI: 13.39–14.25) was more than six-fold that of respondents not living with HIV (2.17%, 95%CI: 2.12–2.23) (estimates not shown in tables), raising from 11.79% (95%CI: 11.23–12.33) in 2010 to 16.15% (95%CI: 15.48–16.82) in 2017 among HIV-diagnosed respondents (Figure 3 and Table 3).

**Table 3:** Syphilis diagnosis in the previous 12 months, number of non-steady male CAI partners, and recency of last STI-screening, overall and in key population groups by survey wave (2010 and 2017) in 31 European countries, European Men-who-have-sex-with-men Internet Survey: EMIS-2010 and EMIS-2017.

	2010											2017							
	Overall		Not taking PrEP^a^		Not eligible for PrEP: HIV+		Sold sex		Overall			Not taking PrEP^b^		Not eligible for PrEP: HIV+		Taking PrEP^c^		Sold sex	
	N	%	N	%	N	%	N	%	N	%		N	%	N	%	N	%	N	%
**Syphilis diagnosis**																			
Yes	3875	2.33	2324	1.52	1551	11.79	494	6.28	5074	4.54		2739	2.84	1859	16.15	453	13.81	468	9.85
No	162,551	97.67	150,944	98.48	11,607	88.21	7373	93.72	106,756	95.46		93,566	97.16	9652	83.85	2828	86.19	4284	90.15
**Number of non-steady male CAI partners**																			
None	118,334	72.79	112,552	75.12	5782	45.39	3541	46.35	64,557	60.20		60,126	65.00	3472	31.75	472	15.28	1436	31.88
1	18,760	11.54	17,619	11.76	1141	8.96	1056	13.82	13,451	12.54		12,224	13.21	929	8.49	213	6.89	563	12.5
2–4	15,831	9.74	13,807	9.22	2024	15.89	1505	19.7	15,434	14.39		12,874	13.92	1888	17.26	593	19.19	1015	22.53
5–-9	3982	2.45	2913	1.94	1069	8.39	498	6.52	5257	4.90		3599	3.89	1174	10.74	459	14.85	436	9.68
10–20	3423	2.11	2050	1.37	1373	10.78	537	7.03	4944	4.61		2551	2.76	1706	15.60	670	21.68	483	10.72
21–30	779	0.48	350	0.23	429	3.37	144	1.88	1371	1.28		503	0.54	610	5.58	256	8.28	172	3.82
31–40	395	0.24	166	0.11	229	1.8	79	1.03	573	0.53		183	0.20	286	2.62	103	3.33	88	1.95
41–50	232	0.14	74	0.05	158	1.24	60	0.79	353	0.33		114	0.12	171	1.56	68	2.20	71	1.58
> 50	826	0.51	293	0.20	533	4.18	220	2.88	1291	1.20		330	0.36	700	6.40	256	8.28	241	5.35
**Recency of last STI-screening previous 12 months**																			
No STI-screening	106,020	67.80	102,726	71.41	3294	26.32	3954	53.75	58,743	54.59		55,715	60.15	2349	21.16	251	7.80	1908	41.77
During previous month	7032	4.50	4980	3.46	2052	16.39	591	8.03	9194	8.54		5763	6.22	2151	19.38	1242	38.60	600	13.13
2–6 months ago	18,242	11.67	14,586	10.14	3656	29.21	1182	16.07	19,696	18.30		14,715	15.89	3795	34.19	1098	34.12	996	21.80
7–12 months ago	13,658	8.73	12,131	8.43	1527	12.2	655	8.90	10,731	9.97		9474	10.23	1088	9.80	104	3.23	370	8.10
Symptomatic STI test	11,415	7.30	9427	6.55	1988	15.88	974	13.24	9244	8.59		6962	7.52	1718	15.48	523	16.25	694	15.19

a Overall sample excluding individuals diagnosed with HIV.

b Overall sample excluding individuals diagnosed with HIV and individuals taking PrEP daily or on demand.

c Taking PrEP daily or on demand.

We observed a dose-response relationship whereby the odds of being diagnosed with syphilis were higher for those who had screened more recently. Compared to those who had not screened within the previous 12 months, the odds ratios of syphilis diagnosis were 25.77 (95%CI: 18.23–36.41), 16.06 (95%CI: 11.63–22.18) and 7.25 (95%CI: 5.37–9.78) for those who had screened asymptomatically within the previous month, 2–6 months, and 7–12 months, respectively (Table 2). For those who had a symptomatic STI test performed, the odds ratio of being diagnosed with syphilis, compared to those who did not screen at all, was 74.82 (95%CI: 51.44–108.81). These estimates accounted for a probability of syphilis diagnosis of 6.10% (95%CI: 5.38–6.82), 3.98% (95%CI: 3.38–), and 1.88% (95%CI: 1.24–2.52) for those who had screened asymptomatically within the previous month, 2–6 months, and 7–12 months, respectively (Figure 2). Those who during the last year had performed a symptomatic STI test had a 14.98% probability of being diagnosed with syphilis. This probability was 0.27% (95%CI: 0.16–0.38) for individuals not screened during the last year. Screening for STI increased between survey waves, particularly among those reporting their last screen was within the previous month (raising from 4.50% (95%CI: 4.40–4.61) in 2010 to 8.54% (95%CI: 8.38–8.71) in 2017) and within the previous 2–6 months (from 11.67% (95%CI: 11.45–11.78) in 2010 to 18.30% (95%CI: 18.03–18.48) in 2017) (Table 1 and Table 3).

Each additional steady male sexual partner (aOR 1.006, 95%CI: 1.001–1.011), non-steady male sexual partner (aOR 1.004, 95%CI: 1.002–1.015), CAI steady male partner (aOR 1.006, 95%CI: 1.001–1.011) and CAI non-steady male partner (aOR 1.008, 95%CI: 1.007–1.009) were associated, on average, with a higher odds of syphilis diagnosis (in more detailed decimal numbers) (Table 2). Figure 2 depicts the association between disaggregated numbers of different types of male sexual partners (using ordinal variables instead of continuous variables as above) with the marginal mean probability of syphilis diagnosis. We observed a substantial increased probability with the CAI non-steady male partners measure (for instance, 3.43% probability of syphilis diagnosis for those reporting more than 50 non-steady male partners vs 7.09% for those reporting more than 50 non-steady male CAI partners), with a strong dose-response relationship, whereby greater numbers of partners were linked to a higher probability of syphilis diagnosis. The mean number of male partners was higher in 2017 compared to 2010, particularly non-steady CAI partners, which more than doubled (Table 1), with doubling numbers occurring among those reporting more than 5 and up to more than 50 non-steady CAI partners (Table 3). The proportion of individuals reporting more than 50 non-steady male CAI partners raised from 0.52% (95%CI: 0.48–0.54) in 2010 to 1.20% (95%CI: 1.14–1.27) in 2017 (Table 3).

Engagement in transactional sex increased the odds of syphilis diagnosis, less among people buying sex (aOR 1.22, 95%CI: 1.21–1.34) than among those selling sex (aOR 1.45, 95%CI: 1.27–1.65) (Table 2); for the latter the marginal probability of syphilis diagnosis was 4.02% (95%CI: 3.39–4.66) vs 3.00% (95%CI: 2.66–3.34) among those not selling sex (Figure 2). Across survey waves, the proportion of syphilis diagnoses among people selling sex (7.62%, 95%CI: 7.16–8.09) was 2.5 times greater than among those who did not (3.01%, 95%CI: 2.94–3.07), increasing from 6.28% (95%CI: 5.74–6.82) in 2010 to 9.85% (95%CI: 9.00–10.70) in 2017 among people selling sex (Figure 3 and Table 3).

Finally, Table 2 shows, first, that respondents of the French questionnaire in 2017 had a higher odds of reporting a syphilis diagnosis (aOR 2.84, 95%CI: 2.37–3.39). Second, individuals recruited via social media had lower odds of syphilis diagnosis compared to individuals recruited via dating apps (aOR 0.81, 95%CI: 0.74–0.90).

### Determinants of the number of non-steady male CAI partners

In 2017, the incidence rate for the count of non-steady male CAI partners was 1.71 (95%CI: 1.67–1.74) times the rate in 2010, after adjusting for multiple potential determinants (Table 4). The expected number of non-steady male CAI partners decreased with educational level in a dose-response relationship (aIRR for high vs low educational level: 0.75, 95%CI: 0.72–0.78; aIRR for mid vs low educational level: 0.88, 95%CI: 0.84–0.92). Compared to employed individuals, unemployed individuals had a higher expected count of non-steady male CAI partners (aIRR 1.17, 95%CI: 1.12–1.22) yet students had a lower expected count (aIRR 0.73, 95%CI: 0.70–0.76). No difference was observed between unemployed individuals and those retired, in long-term sick leave or other (aIRR 1.02, 95%CI: 0.98–1.06). The incidence rate was higher for individuals living in settlements of bigger size (aIRR 1.09, 95%CI: 1.06–1.11) and for individuals born outside the country of residence (aIRR 1.18, 95%CI: 1.14–1.21). Survey participants recruited through social media (aIRR 0.87, 95%CI: 0.84–0.89) or with unknown recruitment (aIRR 0.82, 95%CI: 0.76–0.89) had a lower expected count of non-steady male CAI partners than respondents recruited through dating apps.

**Table 4:** Determinants of change in the incidence rate of reporting a number non-steady CAI partners within the previous 12 months in 31 European countries, European Men-who-have-sex-with-men Internet Survey: EMIS-2010 and EMIS-2017.

	Number of non-steady male CAI partners	
	Adjusted incidence rate ratio	95% confidence interval
**Year**		
2010	reference	
2017	1.705^⁎⁎⁎^	1.667 to 1.743
**Age**	1.047^⁎⁎⁎^	1.041 to 1.053
**Age squared**	1.000^⁎⁎⁎^	0.999 to 1.000
**Educational level**		
Low	reference	
Mid at least upper secondary; 2–5 years post 16	0.881^⁎⁎⁎^	0.844 to 0.920
High first stage of tertiary or more; 6+ years post 16	0.746^⁎⁎⁎^	0.715 to 0.779
**Occupational status**		
Employed full/part/self	1.000	
Unemployed	1.167^⁎⁎⁎^	1.117 to 1.219
Student	0.730^⁎⁎⁎^	0.704 to 0.757
Retired/Long-term sick leave/Other	1.018	0.975 to 1.063
**Settlement size**		
Small town/village <100,000 inhabitants	reference	1.000 to 1.000
Medium/big town ≥100,000 inhabitants	1.089^⁎⁎⁎^	1.064 to 1.114
**Country of birth**		
Born in country of residence	reference	
Born abroad	1.178^⁎⁎⁎^	1.143 to 1.214
**Diagnosed with HIV**		
No	reference	
Yes	3.905^⁎⁎⁎^	3.771 to 4.045
**Recency of last STI-screening or testing**		
No STI-screening previous 12 months	reference	
Last STI-screening during previous month	3.041^⁎⁎⁎^	2.915 to 3.172
Last STI-screening 2–6 months ago	2.014^⁎⁎⁎^	1.953 to 2.077
Last STI-screening 7–12 months ago	1.274^⁎⁎⁎^	1.228 to 1.321
Symptomatic STI test during previous 12 months	3.263^⁎⁎⁎^	3.143 to 3.387
**Paid for sex in the previous 12 months**		
No	reference	
Yes	1.496^⁎⁎⁎^	1.443 to 1.552
**Sold sex in the previous 12 months**		
No	reference	
Yes	4.388^⁎⁎⁎^	4.193 to 4.593
**Knowledge about HIV undetectable equals untransmissible (U=U)**		
I didn't know/understand/believe/wasn't sure	1.000	
I knew this already	1.625^⁎⁎⁎^	1.590 to 1.661
**CAI partners based on HIV-serosorting in the previous 12 months**		
No	reference	
Yes	1.686^⁎⁎⁎^	1.627 to 1.747
**Survey recruitment source**		
Dating apps (Romeo, Grindr, Hornet, other dating apps/websites)	reference	
Social media (Facebook, Twitter, Instagram, other)	0.868^⁎⁎⁎^	0.843 to 0.894
Unknown tracking code	0.822^⁎⁎⁎^	0.764 to 0.885
Log-transformed overdispersion parameter	4.672^⁎⁎⁎^	4.627 to 4.718
Country random intercepts	1.028^⁎⁎^	1.011 to 1.045
Number of individuals	203467	

* *p* < 0.05, ^⁎⁎^*p* < 0.01, ^⁎⁎⁎^*p* < 0.001; Robust standard errors adjusted by country; CAI, condomless anal intercourse.

**Figure 4:**
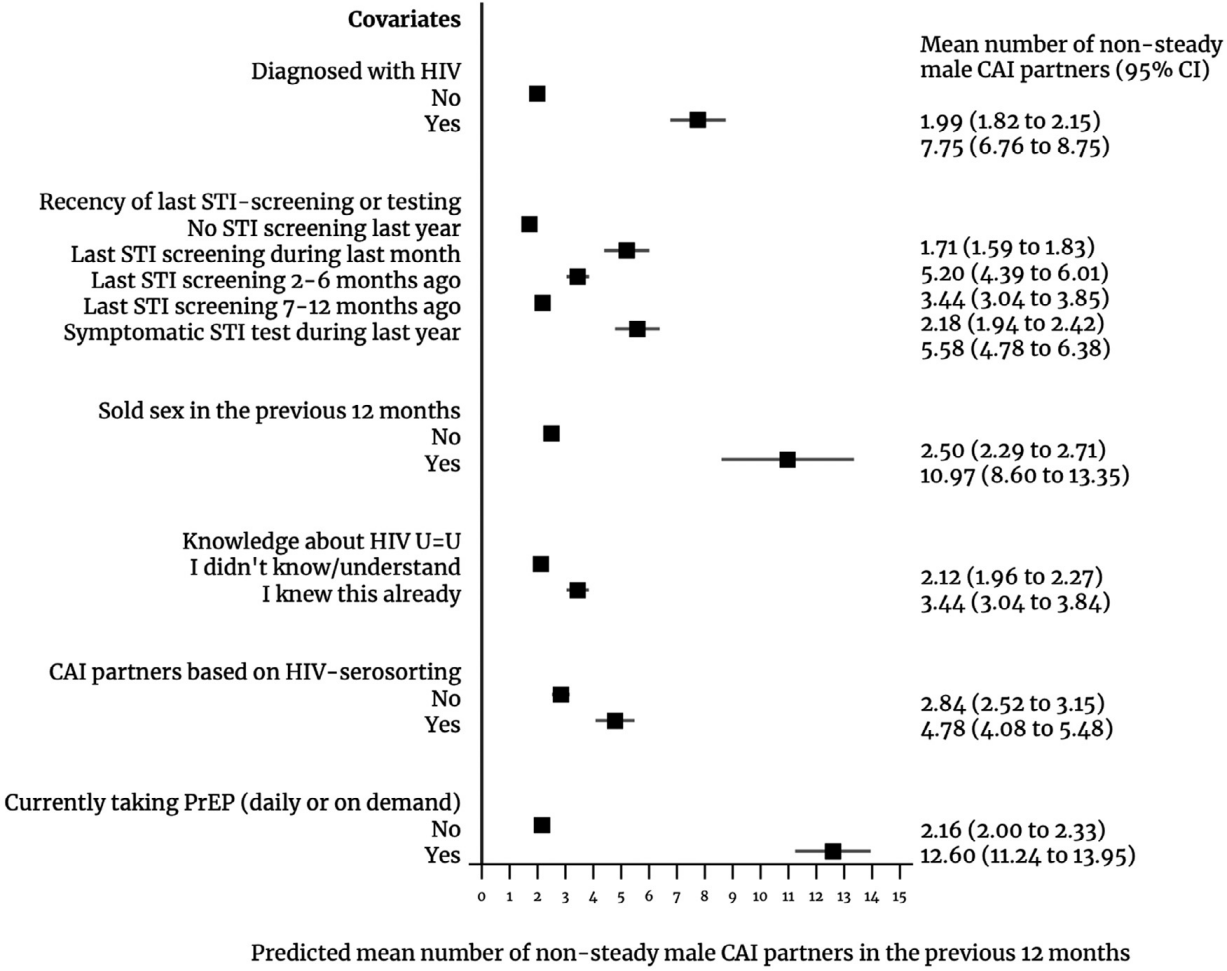
Predicted number of non-steady male CAI partners in the previous 12 months for selected covariates (marginal predicted mean number) in 31 European countries, European Men-who-have-sex-with-men Internet Survey: EMIS-2010 and EMIS-2017. CAI, condomless anal intercourse). Results for covariate measuring PrEP use are only for the year 2017 and the estimates for this covariate are based on a sample size of only 30 countries as in one country (Latvia) there were no PrEP users.

We observed the greatest impact on the number of non-steady male CAI partners to be associated with HIV diagnosis and behavioural and interventional factors. We found men with diagnosed HIV had an incidence rate for the number of non-steady male CAI partners 3.91 (95%CI: 3.77–4.05) times that of those without diagnosed HIV (Table 4). The marginal predicted number of non-steady male CAI partners was 7.75 (95%CI: 6.76–8.75) among people living with diagnosed HIV vs 1.99 (95%CI: 1.82–2.15) among those not diagnosed with HIV (Figure 4). We estimated the reported number of non-steady CAI partners among people living with HIV rose from 10.16 (95%CI: 9.71–10.62) in 2010 to 14.96 (95%CI: 14.38–15.55) in 2017 (results not in tables, disaggregated by bands in Table 3).

More recent STI-screening was also associated with a higher number of non-steady male CAI partners: compared with no screening in the previous 12 months, the incidence rate for the count of non-steady male CAI partners was 3.04 (95%CI: 2.92–1.72), 2.01 (95%CI: 1.95–2.08), and 1.27 (95%CI: 1.23–1.32) times, respectively, for those who had screened asymptomatically within the previous month, 2–6 months, and 7–12 months (Table 4). For these, the predicted mean number of non-steady male CAI partners were 5.20 (95%CI: 4.39–6.01), 3.44 (95%CI: 3.04–3.85), and 2.18 (95%CI: 1.94–2.42), respectively (Figure 4). Those who had a test with symptoms had an incidence rate 3.26 (95%CI: 3.14–3.39) times that of those with no screening in the previous 12 months and a predicted mean number of non-steady male CAI partners of 5.58 (95%CI: 4.78–6.38) (Table 4 and Figure 4).

Engagement in transactional sex was also linked with a higher number of non-steady male CAI partners, particularly among those selling sex (aIRR 4.39, 95%CI: 4.19–4.59), for whom the predicted mean number of partners was 10.97 (95%CI: 8.60–13.35) vs 2.50 (95%CI: 2.29–2.71) partners for those not selling sex. For people selling sex, the number of non-steady male CAI partners rose between waves for all partner number categories above 10 partners or more, almost doubling for more than 50 partners (Table 3). Those buying sex had an incidence rate 1.50 (95%CI: 0.63–1.29) times higher than that of those not paying for sex.

Individuals aware that an HIV undetectable viral load makes the virus untransmissible (U=U), had a higher expected count of non-steady male CAI partners (aIRR 1.63, 95%CI: 1.59–1.66) than those not aware of it (Table 4), whereby the mean predicted number of non-steady male CAI partners for those with knowledge about U=U was 3.44 (95%CI: 3.04–3.84) vs 2.12 for those without it (95%CI: 1.96–2.27) (Figure 4). Individuals practicing HIV-serosorting also had a higher expected number of non-steady male CAI partners (aIRR 1.69, 95%CI: 1.63–1.75) (Table 4), with a predicted number of non-steady male CAI partners of 4.78 (95%CI: 4.08–5.48) vs 2.84 (95%CI: 2.52–3.15) among those not serosorting (Table 4 and Figure 4).

### PrEP, syphilis, and CAI

In 2017, PrEP use (daily or on demand) was linked to having substantially higher numbers of non-steady male CAI partners: individuals currently using PrEP had an incidence rate for the count of non-steady male CAI partners 7.90 (95%CI: 6.99–8.94) times that of those who were not using PrEP (Table 5), corresponding to a predicted mean number of partners of 12.60 (95%CI: 11.24–13.95) and 2.16 (95%CI: 2.00–2.33), respectively (Figure 4). This effect did not have a substantial reduction after adjusting for the recency of last screening (aIRR 5.82, 95%CI: 5.29–6.41). In 2017, more than a third (38.60%, 95%CI: 36.93–40.29) of people using PrEP screened for STIs within the previous month, compared to only 6.22% (95%CI: 6.07–6.38) of those who did not take PrEP, and 19.38% (95%CI: 18.65–20.12) of people living with diagnosed HIV; almost two thirds of PrEP users had screened for STIs within the previous six months (Table 3).

**Table 5:** Association of PrEP use (currently using PrEP daily or on demand vs not currently using PrEP) with the incidence rate ratio of condomless anal intercourse (CAI) non-steady partners within the previous 12 months, and odds ratio of syphilis diagnosis, in 30 European countries, European Men-who-have-sex-with-men Internet Survey: EMIS-2010 and EMIS-2017.

		Association of PrEP use with the number of non-steady CAI partners and probability of syphilis diagnosis		
		Adjusting for sociodemographic and behavioural variables^a^ (except STI-screening)	Adjusting for sociodemographic and behavioural variables^a^ (including STI-screening)	Adjusting for sociodemographic and behavioural variables^a^ (including STI-screening and number of non-steady male CAI partners)
**Incidence rate ratio of the number of non-steady male CAI partners in the previous 12 months**	Not currently taking PrEP	reference	reference	
	PrEP daily or on demand	7.902^⁎⁎⁎^	5.821^⁎⁎⁎^	
		(6.987 to 8.936)	(5.289 to 6.407)	N/A
		*N = 77,203*	*N = 74,309*	
**Odds ratio of syphilis diagnosis in the previous 12 months**^b^	Not currently taking PrEP	reference	reference	reference
	PrEP daily or on demand	3.018^⁎⁎⁎^	1.610^⁎⁎^	1.199
		(2.298 to 3.962)	(1.198 to 2.164)	(0.894 to 1.607)
		*N = 79,416*	*N = 76,535*	*N = 73,456*

*p < 0.05, ^⁎⁎^*p* < 0.01, ^⁎⁎⁎^*p* < 0.001; 95% confidence intervals in brackets; robust standard errors adjusted by country; CAI, condomless anal intercourse.

Note: sample size includes only responses for 2017 and eligible PrEP users (*i.e*., HIV-diagnosed respondents are excluded from the sample); sample includes only 30 countries as in one country (Latvia) there were no PrEP users.

a Models adjusted for all covariates shown in Table 2.

b Models adjusted for language of questionnaire.

In 2017, the proportion of respondents using PrEP reporting a syphilis diagnosis (13.81%, 95%CI: 12.67–15.03) was almost five-fold that of respondents not using PrEP (2.84%, 95%CI: 2.74–2.95) (Figure 2 and Table 3). PrEP use was linked to a greater odds of syphilis diagnosis (aOR 3.08, 95%CI: 2.30–3.96), after adjusting for sociodemographic factors, HIV diagnosis, and engagement in transactional sex. To assess the role of STI-screening and number of non-steady male CAI partners as effect mediators of the association between PrEP use and syphilis diagnosis, we controlled for these factors observing that the effect size substantially reduced and that the association did not hold (aOR 1.20, 95%CI: 0.84–1.61).

### Robustness checks

We performed a series of robustness checks by fitting alternative models (multivariable linear probability and multivariable linear regression models) and by testing our model specifications by removing cases with discrepant data (Supplemental data: Appendix 2-6). In each case, our results did not substantially change.

## Discussion

Our study shows that self-reported syphilis diagnoses have risen markedly among MSM responding to EMIS. The rise was across all European countries, except for Luxembourg, which is likely to be attributable to non-captured sampling effects. Syphilis diagnoses were strongly associated with living with diagnosed HIV, taking PrEP, and selling sex, and the rise in syphilis diagnoses has disproportionately impacted HIV-diagnosed MSM and MSM sex workers (no longitudinal data on PrEP users can be calculated due to the non-availability of PrEP in 2010). Major determinants associated with increased syphilis diagnoses were more recent STI-screening uptake and increased number of non-steady male CAI partners, both higher in 2017 compared to 2010, and both variables mediating the association between PrEP use and higher chance of syphilis diagnosis. MSM who were PrEP users, HIV-diagnosed, or sex workers reported the highest rates of STI-screening uptake and number of non-steady male CAI partners (confirming results of a previous analysis),^16^ which may explain the triple concentration of syphilis in these three population subgroups.

Behavioural changes associated with the syphilis epidemics may partly be due to the evolving consensus on the effectiveness of treatment as prevention: undetectable equals untransmissible and PrEP altering the need for condom in HIV serodiscordant sexual relationships. While we found the number of non-steady male CAI partners to be a mediator of the association between PrEP use and higher odds of syphilis diagnosis, this study was not able to discern whether individuals using PrEP were already having higher numbers of non-steady male CAI partners before initiating PrEP. If this was the case, there has been correct population targeting of the intervention, considering that CAI is a major reason these individuals seek, and clinicians recommend, PrEP use. Nor can it show whether use of PrEP led to increases in number of CAI partners. Further research could longitudinally investigate behaviour changes following PrEP use and their link to increases in syphilis incidence. Recent STI-screening was a key factor of syphilis diagnoses. Individuals screened for STIs more recently reported higher numbers of non-steady male CAI partners, indicating more syphilis-screening among MSM with more risky sexual behaviour, such as PrEP users, HIV-diagnosed individuals, and people selling sex, with higher numbers of non-steady male CAI partners. Medical monitoring of people living with HIV and, even more so, in PrEP users include sexual health counselling and routine STI-screening, which can contribute to diagnosing and treating STIs in highly exposed MSM.^26^ Many European countries already recommend three-monthly syphilis-screening in PrEP users, *e.g.* the United Kingdom.^29^ However, restricting more regular syphilis-screening in MSM to individuals already included in clinical follow-up (such as MSM diagnosed with HIV or PrEP users) might not be enough to control the syphilis epidemic in MSM. Some countries, therefore, explicitly recommend biannual syphilis-screening in multi-partners MSM.^30^^,^^31^

Our study has several limitations. The study sample is likely not representative of all MSM. The online recruitment strategy over-samples more sexually active MSM, those who use the internet and/or dating apps to meet sexual partners.^32^ Nevertheless, estimates of national HIV prevalence from EMIS-2010 were strongly correlated with existing estimates based on biological measurement and modelling studies using surveillance data.^33^

Second, there are likely other determinants of syphilis acquisition not included in our models, such as sexual locations (*e.g.,* house-parties, saunas) and use of typical chemsex drugs,^17^^,^^18^ or that were not measured in both EMIS surveys (*e.g.,* group sex, combining sex and drugs; and anti-LGBT structural stigma).^34^

Third, potential response biases could understate or overstate individual risk on several dimensions, including the total number of male sexual and CAI partners, and/or paying for or selling sex. Measurement bias can also arise in self-reports where respondents omit or incorrectly report the time of syphilis diagnosis. Stigma, social desirability and recall bias may all play a part. The anonymous online format of the survey may have minimized some of these biases. Measurement error makes it harder to detect statistical relationships should they actually exist, as a result biasing our estimated effect sizes in a conservative direction.

Fourth, there was a non-trivial proportion of cases with discrepant data, which involved anomalous or inconsistent reporting of age and partner numbers. However, their inclusion or exclusion did not alter the study's main findings.

Fifth, data on syphilis diagnosis does not include information on type of diagnostic assay. However, between survey waves, there were no major advances in syphilis diagnostic tests, so that any potential bias is likely to be non-differential with regard to our research question.

Clinical implications derived from our results are that MSM disclosing multiple CAI partners should be offered syphilis-screening due to their clearly increased risk and the serious possible sequelae of syphilis. Guidelines for people using PrEP include frequent syphilis-screening, for which our finding that over a third of PrEP users screened within the previous month provides support for the feasibility of implementing a regular HIV- and syphilis-screening approach. Further, community-based education in MSM communities is needed to increase knowledge of and social norms for syphilis-screening. Approaches to foster syphilis-screening, such as online tools for risk assessment, home-sampling, and free at-point-of use tests for men without a previous history of syphilis, along with tools for partner notification to interrupt transmission chains, could be considered as additional combined interventions for national syphilis control and elimination strategies.

## Contributors

CRediT author statement (https://www.elsevier.com/authors/policies-and-guidelines/credit-author-statement)

Conceptualization: A.M.L., D.S., T.N., J.C.S.

Methodology: A.M.L., D.S., J.C.S., A.J.S.

Software: A.M.L.

Validation: A.M.L., D.S., A.J.S.

Formal analysis: A.M.L.

Investigation – literature review: A.M.L., D.S., J.C.S.

Investigation – data collection: A.J.S., F.H., U.M., R.N.W., A.D., L.H., A.V.

Resources: A.M.L., A.J.S

Data curation: A.J.S.

Writing – original draft: A.M.L., D.S., J.C.S.

Writing – review and editing: all authors

Visualisation: A.M.L.

Supervision: A.M.L., D.S., J.C.S., A.J.S.

Funding acquisition for this paper: J.C.S., D.S.

## Data sharing statement

The EMIS-2017 dataset used for this analysis has been obtained from the London School of Hygiene and Tropical Medicine under a data transfer agreement that prohibits to sharing the dataset publicly. Although we cannot make study data publicly accessible at the time of publication, all authors commit to make the data underlying the findings of the study available in compliance with the Lancet Data Availability Policy. Data requests should be addressed to the London School of Hygiene and Tropical Medicine Research Operations Office Data Management Lead: alex.hollander@lshtm.ac.uk, the last author (axel.schmidt@lshtm.ac.uk), and the Principal Investigator of EMIS-2017 (Peter.Weatherburn@lshtm.ac.uk). Individuals requesting data should present their research objective(s) and enclose a list of requested variables. To protect the confidentiality of participants, data sharing is contingent upon appropriate data handling and good scientific practice by the person requesting the data and should furthermore be in accordance with all applicable local requirements. The London School of Hygiene and Tropical Medicine administrative offices are located at Keppel Street, London WC1E 7HT, United Kingdom.

## Declaration of interests

We declare no competing interests.

## Appendix Supplementary materials

Image, application 1
