# Supplementary material for: Social and behavioural determinants of syphilis: Modelling based on repeated cross-sectional surveys from 2010 and 2017 among 278,256 men who have sex with men in 31 European countries
Source: Lancet Reg Health Eur. 2022 Aug 9;22:100483. doi: 10.1016/j.lanepe.2022.100483 (PMC9382326; doi:10.1016/j.lanepe.2022.100483)
Supplement: Supplementary file 1 [file mmc1.docx]

**Supplemental data**

*Table of contents*

Appendix 1. EMIS survey details: additional information for recruitment and included variables

Appendix 2. Determinants of change in the probability of reporting a syphilis diagnosis within the previous 12 months in 31 European countries, European Men-who-have-sex-with-men Internet Survey: EMIS-2010 and EMIS-2017

Appendix 3. Change in the probability of reporting a syphilis diagnosis within the previous 12 months by number of steady and non-steady partners, and number of steady and non-steady condomless anal intercourse (CAI) partners, all within the previous 12 months, in 31 European countries, European Men-who-have-sex-with-men Internet Survey: EMIS-2010 and EMIS-2017

Appendix 4. Determinants of change in the number of non-steady male CAI partners within the previous 12 months in 31 European countries, European Men-who-have-sex-with-men Internet Survey: EMIS-2010 and EMIS-2017

Appendix 5. Association of PrEP use (currently using PrEP daily or on demand vs not currently using PrEP) with number of non-steady male CAI partners within the previous 12 months, and probability of syphilis diagnosis, in 30 European countries, European Men-who-have-sex-with-men Internet Survey: EMIS-2017

Appendix 6. Determinants of change in the probability of syphilis diagnosis in the previous 12 months in 31 European countries, European Men-who-have-sex-with-men Internet Survey: EMIS-2010 and EMIS-2017: Testing discrepant cases

Appendix 1. EMIS survey details: additional information for recruitment and included variables

***Data source:***

EMIS was available in 25 languages in 2010 and 33 languages in 2017. The 2017 wave differed principally from 2010 by including the use of smartphone apps (65.8% of the 2017 analytic sample), which had become commonplace across all countries surveyed. Recruitment took place through promotion via supportive organisations within the EMIS network (national/trans-national, civil society, HIV/LGBT groups, *etc.*), accounting for 20.7% of recruits in EU/EFTA countries in 2010, and for 15.6% of recruits in 2017—often using social networking sites (Facebook, Twitter and Instagram). In both waves, however, the majority of recruits came via direct message invitations and advertising on geo-spatial dating websites and smartphone applications (79.0% in 2010, 79.6% in 2017, the most prominent being PlanetRomeo in both waves, the second most prominent in 2017 being Grindr with 21.4%). The 2017 response rate in EU/EFTA countries was 6.6 per 10,000 men aged 15–65, ranging from 3.0 in Poland to 20.1 in Malta. The survey designers suggest that this figure roughly translates to 2.3% of all MSM in EU/EFTA countries. Further details about the data are provided in the EMIS design and methods papers.^24,25^ More information can also be found in the EMIS website: [www.emis2017.eu](http://www.emis2017.eu).

***Discrepant data:***

Discrepancies in data arise if answers to two or more questions are logically inconsistent for any of three key variables: age, steady male partners and non-steady partners, each with seven, five and six possible inconsistencies respectively; if a case had at least one inconsistency in any of these three areas, it was flagged as discrepant (n=35,859, 12.89% of the sample). In robustness checks, discrepant cases were excluded.

***Variables:***

**Educational level:** In EMIS-2010, the classification for the variable education was done according to levels of the International Classification of Education (ISCED) from 1997, where ISCED 1 corresponds to the lowest level of education of the scale (primary education) and ISCED 6 corresponds to the higher level of education (second stage tertiary education, *e.g*., PhD). In EMIS-2017, we instead asked for the years spent in full-time education since the age of 16. How these years can be converted into ISCED categories differs across time and across countries. For the sake of comparability, we consulted a manual published by the Organisation for Economic Co-development and Development (OECD), to convert the new measure into ISCED levels (Organisation for Economic Co-operation and Development: *Classifying Educational Programmes. Manual for ISCED-97 Implementation in OECD Countries*. Paris, 1999). Very broadly speaking, 2–5 years of education beyond 16 years correspond to an ISCED-97 level three (upper secondary education); 6 years or more correspond to an ISCED-97 level five (first stage of tertiary education), and more than 10 years correspond to an ISCED-97 level six (second stage of tertiary education). In this study educational categories low, mid and high correspond to the following 1997 ISCED levels: Low: ISCED 1–2; Mid: ISCED 3–4; and High: ISCED 5–6. Further details about the data are provided in the EMIS design and methods papers.^24,25^ More information can also be found in the EMIS website: [www.emis2017.eu](http://www.emis2017.eu).

**Number of steady and non-steady [CAI] partners:** These variables were collected as discrete with intervals for higher number of partners and truncated for the category at the top end, i.e. 1, 2, 3… 8, 9, and 10+ (steady male partners), or 1, 2, 3…8, 9, 10, 11–20, 21–30, 31–40, 41–50, and more than 50 (non-steady male partners). Numeric substitutes for each of the interval and truncated categories are based on findings from a meta-analysis.^27^ They were rounded to the nearest decimal when used in hierarchical negative binomial models. Nonetheless, estimates of the impact of these variables on risk of syphilis diagnosis are also provided using the variables in their original categorical format (see Figure 3).

***Sub-optimal translation syphilis diagnosis question in French questionnaire and adjustment:***

The sub-optimal translation of the syphilis diagnosis question in the French language questionnaire in 2017 occurred because the way in which it was asked may have made some respondents think that they were being surveyed about whether they had a syphilis diagnostic test performed instead of being diagnosed with syphilis. Therefore, although technically correct, this wording in the French questionnaire has probably inflated the number of people reporting a positive answer to the question. The exact lead-in question was *“Have you ever been diagnosed with syphilis?”*, and in French “*Avez­vous déjà eu un diagnostic de syphilis*?” (2017) vs. “*Avez­vous déjà eu un diagnostic positif de syphilis?”* (2010)

A total of 10.8% of the study sample used the French version of the questionnaire in 2017. The countries mostly affected were France (with 93.3% of respondents using the French questionnaire), Belgium (35.9%), Luxembourg (27.2%), and Switzerland (19.2%).

We adjusted all statistical analyses for this translation using a dichotomous variable to indicate whether the questionnaire of 2017 language was French as in 2010 the wording was not misleading. Further details about the data source are provided in the EMIS design and methods papers.^24,25^ More information can also be found in the EMIS website: [www.emis2017.eu](http://www.emis2017.eu)

Appendix 2. Determinants of change in the probability of reporting a syphilis diagnosis within the previous 12 months in 31 European countries, European Men-who-have-sex-with-men Internet Survey: EMIS-2010 and EMIS-2017

|  | **Model 1**  **Probability of syphilis diagnosis** | | **Model 2**  **Probability of syphilis diagnosis** | |
| --- | --- | --- | --- | --- |
|  | Adjusted beta coefficient | 95% Confidence Interval | Adjusted beta coefficient | 95% Confidence Interval |
| **Year** |  |  |  |  |
| 2010 | reference |  | reference |  |
| 2017 | 0.0137^***^ | 0.0112 to 0.0162 | 0.0027 | -0.0003 to 0.0056 |
|  |  |  |  |  |
| **Questionnaire language** |  |  |  |  |
| Other than French | reference |  | reference |  |
| French | 0.0763^***^ | 0.0538 to 0.0988 | 0.0671^***^ | 0.0495 to 0.0847 |
|  |  |  |  |  |
| **Age** |  |  | 0.0008^***^ | 0.0005 to 0.0011 |
| **Age squared** |  |  | -0.0001^***^ | -0.0001 to -0.0001 |
|  |  |  |  |  |
| **Educational level** |  |  |  |  |
| Low |  |  | reference |  |
| Mid (at least upper secondary; 2–5 years post 16) |  |  | -0.0046^***^ | -0.0071 to -0.0021 |
| High (first stage of tertiary or more; 6+ years post 16) |  |  | -0.0106^***^ | -0.0143 to -0.0069 |
|  |  |  |  |  |
| **Occupational status** |  |  |  |  |
| Employed full/part/self |  |  | reference |  |
| Unemployed |  |  | 0.0033^**^ | 0.0010 to 0.0055 |
| Student |  |  | -0.0050^***^ | -0.0062 to -0.0039 |
| Retired/Long-term sick leave/Other |  |  | -0.0048^**^ | -0.0077 to -0.0019 |
|  |  |  |  |  |
| **Settlement size** |  |  |  |  |
| Small town/village <100,000 inhabitants. |  |  | reference |  |
| Medium/big town ≥100,000 inhabitants |  |  | -0.0020 | -0.0047 to 0.0006 |
|  |  |  |  |  |
| **Country of birth** |  |  |  |  |
| Born in country of residence |  |  | reference |  |
| Born abroad |  |  | 0.0051^**^ | 0.0014 to 0.0089 |
|  |  |  |  |  |
| **Diagnosed with HIV** |  |  |  |  |
| No |  |  | reference |  |
| Yes |  |  | 0.0647^***^ | 0.0592 to 0.0702 |
|  |  |  |  |  |
| **Recency of last screening or testing** |  |  |  |  |
| No STI-screening previous 12 months |  |  | reference |  |
| Last STI-screening during previous month |  |  | 0.0595^***^ | 0.0445 to 0.0746 |
| Last STI-screening 2–6 months ago |  |  | 0.0306^***^ | 0.0198 to 0.0414 |
| Last STI-screening 7–12 months ago |  |  | 0.0093^*^ | 0.0016 to 0.0170 |
| Symptomatic STI test during previous 12 months |  |  | 0.1661^***^ | 0.1370 to 0.1952 |
|  |  |  |  |  |
| **Number of steady male sexual partners in the previous 12 months** |  |  | 0.0002 | -0.0001 to 0.0004 |
|  |  |  |  |  |
| **Number of non-steady male sexual partners in the previous 12 months** |  |  | 0.0002^***^ | 0.0001 to 0.0002 |
|  |  |  |  |  |
| **Number of steady male CAI partners in the previous 12 months** |  |  | 0.0008^*^ | 0.0002 to 0.0015 |
|  |  |  |  |  |
| **Number of non-steady male CAI partners in the previous 12 months** |  |  | 0.0015^***^ | 0.0013 to 0.0016 |
|  |  |  |  |  |
| **Paid for sex in the previous 12 months** |  |  |  |  |
| No |  |  | reference |  |
| Yes |  |  | 0.0058^**^ | 0.0020 to 0.0097 |
|  |  |  |  |  |
| **Sold sex in the previous 12 months** |  |  |  |  |
| No |  |  | reference |  |
| Yes |  |  | 0.0127^***^ | 0.0058 to 0.0197 |
|  |  |  |  |  |
| Number of individuals | 278,256 |  | 234,719 |  |
| *R*^2^ | 0.02 |  | 0.14 |  |

^*^ *p* < 0.05, ^**^ *p* < 0.01, ^***^ *p* < 0.001; robust standard errors adjusted by country; CAI: condomless anal intercourse. *Note*: In this model, the beta coefficient translates directly to the change in percent points (ppt) after converting the probability (in a scale from 0 to 1) to a percentage (in a scale from 0 to 100).

Appendix 3. Change in the probability of reporting a syphilis diagnosis within the previous 12 months by number of steady and non-steady partners, and number of steady and non-steady condomless anal intercourse (CAI) partners, all within the previous 12 months, in 31 European countries, European Men-who-have-sex-with-men Internet Survey: EMIS-2010 and EMIS-2017


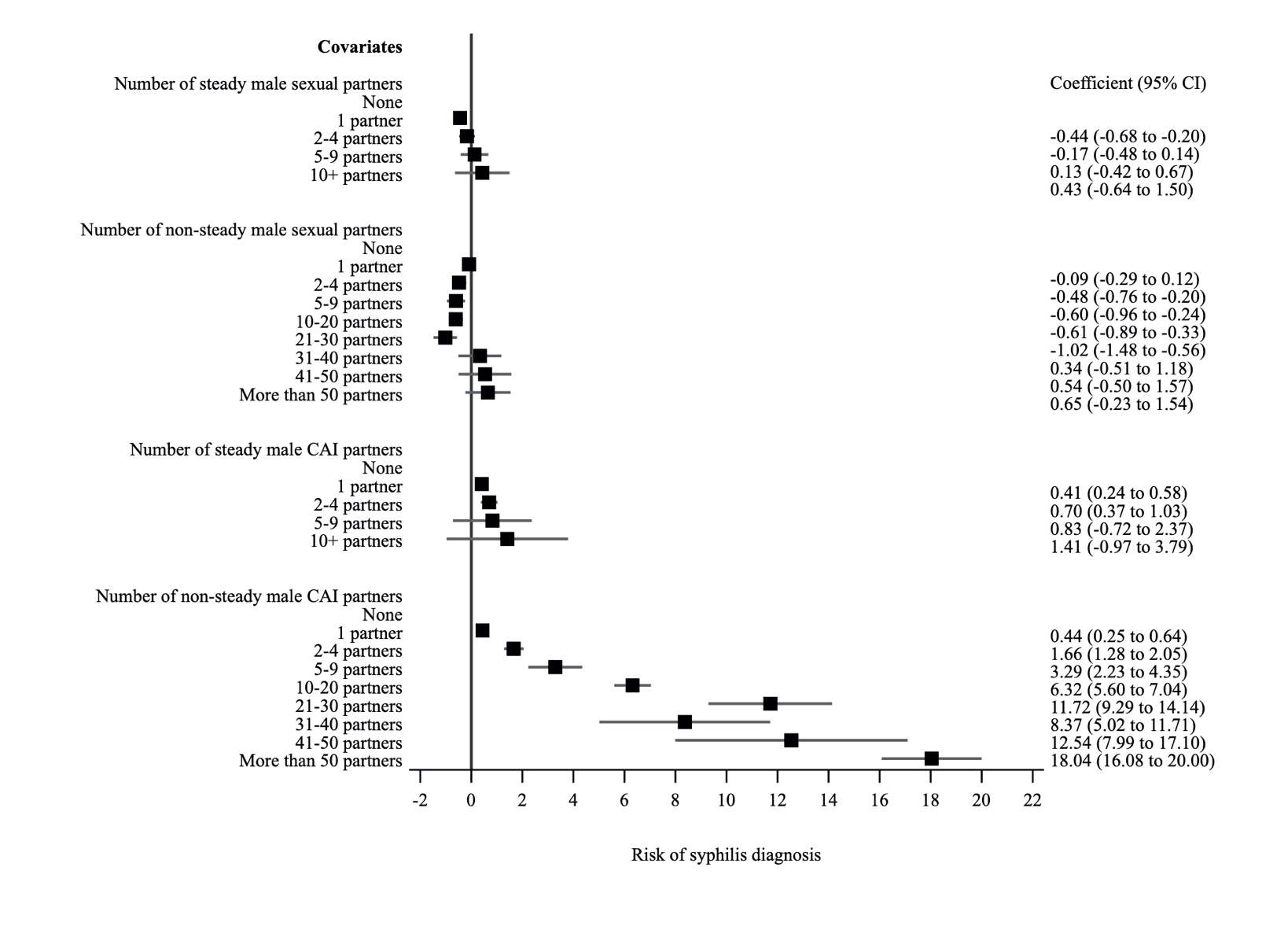


Note: Model adjusted for all covariates in Appendix 2, Model 2. Variables for number of steady and non-steady sexual partners and number of condomless steady and non-steady sexual partners are presented here by categories instead of as continuous variables like in Appendix 2.

Appendix 4. Determinants of change in the number of non-steady male CAI partners within the previous 12 months in 31 European countries, European Men-who-have-sex-with-men Internet Survey: EMIS-2010 and EMIS-2017

|  | **Number of non-steady male CAI partners** | |
| --- | --- | --- |
|  | Adjusted beta coefficient | 95% Confidence Interval |
| **Year** |  |  |
| 2010 | reference |  |
| 2017 | 1.62^***^ | 1.37 to 1.87 |
|  |  |  |
| **Age** | 0.13^***^ | 0.08 to 0.19 |
| **Age squared** | -0.01^***^ | -0.01 to -0.01 |
|  |  |  |
| **Educational level** |  |  |
| Low | reference |  |
| Mid at least upper secondary; 2–5 years post 16 | 0.05 | -0.17 to 0.27 |
| High first stage of tertiary or more; 6+ years post 16 | -0.28^*^ | -0.52 to -0.04 |
|  |  |  |
| **Occupational status** |  |  |
| Employed full/part/self | reference |  |
| Unemployed | 0.24 | -0.05 to 0.53 |
| Student | -0.33^*^ | -0.58 to -0.07 |
| Retired/Long-term sick leave/Other | -0.02 | -0.23 to 0.19 |
|  |  |  |
| **Settlement size** |  |  |
| Small town/village <100,000 inhabitants | reference |  |
| Medium/big town ≥100,000 inhabitants | 0.36^***^ | 0.19 to 0.52 |
|  |  |  |
| **Country of birth** |  |  |
| Born in country of residence | reference |  |
| Born abroad | 0.24 | -0.11 to 0.60 |
|  |  |  |
| **Diagnosed with HIV** |  |  |
| No | reference |  |
| Yes | 9.58^***^ | 8.34 to 10.81 |
|  |  |  |
| **Recency of last STI-screening or testing** |  |  |
| No STI-screening previous 12 months | reference |  |
| Last STI-screening during previous month | 4.29^***^ | 3.38 to 5.20 |
| Last STI-screening 2–6 months ago | 1.39^***^ | 1.13 to 1.64 |
| Last STI-screening 7–12 months ago | -0.25 | -0.55 to 0.04 |
| Symptomatic STI test during previous 12 months | 4.41^***^ | 3.69 to 5.13 |
|  |  |  |
| **Paid for sex in the previous 12 months** |  |  |
| No | reference |  |
| Yes | 0.96^***^ | 0.63 to 1.29 |
|  |  |  |
| **Sold sex in the previous 12 months** |  |  |
| No | reference |  |
| Yes | 6.18^***^ | 4.97 to 7.39 |
|  |  |  |
| **Knowledge about HIV undetectable equals untransmissible (U=U)** |  |  |
| I didn't know/understand/believe/wasn't sure | reference |  |
| I knew this already | 1.24^***^ | 1.04 to 1.45 |
|  |  |  |
| **Serosorting in the previous 12 months*** |  |  |
| No | reference |  |
| Yes | -0.17 | -0.61 to 0.28 |
|  |  |  |
| Number of individuals | 203,467 |  |
| *R*^2^ | 0.11 |  |

^*^ *p* < 0.05, ^**^ *p* < 0.01, ^***^ *p* < 0.001; robust standard errors adjusted by country; CAI: condomless anal intercourse. * Non-steady male CAI partners based on HIV-serosorting in the previous 12 months.

Appendix 5. Association of PrEP use (currently using PrEP daily or on demand vs not currently using PrEP) with number of non-steady male CAI partners within the previous 12 months, and probability of syphilis diagnosis, in 30 European countries, European Men-who-have-sex-with-men Internet Survey: EMIS-2017

|  |  | **Association of PrEP use with the number of non-steady CAI partners and probability of syphilis diagnosis** | | |
| --- | --- | --- | --- | --- |
|  |  | Adjusting for sociodemographic and behavioural variables^a^ (except STI-screening) | Adjusting for sociodemographic and behavioural variables^a^ (including STI-screening) | Adjusting for sociodemographic and behavioural variables^a^ (including STI-screening and number of non-steady CAI partners) |
|  |  |  |  |  |
| **Number of non-steady male CAI partners in the previous 12 months** | Not currently taking PrEP | reference | reference |  |
|  | PrEP daily or on demand | 17.51^***^ | 16.53^***^ |  |
|  |  | (16.11 to 18.90) | (15.27 to 17.78) | N/A |
|  |  | *N=77,203* | *N=74,309* |  |
|  |  |  |  |  |
| **Probability of syphilis diagnosis in the previous 12 months** ^b^ | Not currently taking PrEP | reference | reference | reference |
|  | PrEP daily or on demand | 0.0861^***^ | 0.0530^***^ | 0.0285^***^ |
|  |  | (0.0615 to 0.1107) | (0.0403 to 0.0657) | (0.0168 to 0.0403) |
|  |  | *N=79,416* | *N=76,535* | *N=73,456* |
|  |  |  |  |  |

^*^ *p* < 0.05, ^**^ *p* < 0.01, ^***^ *p* < 0.001; 95% confidence intervals in brackets; robust standard errors adjusted by country; CAI: condomless anal intercourse.

Note: sample size includes only responses for 2017 and eligible PrEP users (*i.e*., HIV-diagnosed respondents are excluded from the sample); sample includes only 30 countries as in one country (Latvia) there were no PrEP users.

(^a^) models adjusted for all covariates shown in Table 2;

(^b^) models adjusted for language of questionnaire.

Appendix 6. Determinants of change in the probability of syphilis diagnosis in the previous 12 months in 31 European countries, European Men-who-have-sex-with-men Internet Survey: EMIS-2010 and EMIS-2017: Testing discrepant cases

|  | Including all cases | Excluding discrepant cases | Only discrepant cases |
| --- | --- | --- | --- |
| ***Year*** |  |  |  |
| 2010 | reference | reference | reference |
| 2017 | 0.0137^***^ | 0.0127^***^ | 0.0242^***^ |
|  | (0.0112 to 0.0162) | (0.0102 to 0.0152) | (0.0153 to 0.0331) |
|  |  |  |  |
| Number of individuals | 278,256 | 242,397 | 35,859 |
| *R*^2^ | 0.02 | 0.02 | 0.02 |

^*^ *p* < 0.05, ^**^ *p* < 0.01, ^***^ *p* < 0.001; 95% confidence intervals in brackets; robust standard errors adjusted by countries.
Models are adjusted for survey wave, language of questionnaire, and country fixed effects.

*Discrepant data:*

Discrepancies in data arise if answers to two or more questions are logically inconsistent for any of three key variables: age, steady male partners and non-steady partners, each with seven, five and six possible inconsistencies respectively; if a case had at least one inconsistency in any of these three areas, it was flagged as discrepant (n=35,859, 12.89% of the analytic sample).
